# Association between nutritional status and food texture levels in older patients with stroke-related sarcopenia in the subacute phase: a retrospective cross-sectional study

**DOI:** 10.3389/fnut.2025.1674577

**Published:** 2025-10-03

**Authors:** Momoko Sakurai, Norikazu Hishikawa, Koshiro Sawada, Suzuyo Ohashi, Hiroshi Maeda, Yasuo Mikami

**Affiliations:** ^1^Department of Rehabilitation Medicine, Graduate School of Medical Science, Kyoto Prefectural University of Medicine, Kyoto, Japan; ^2^Department of Rehabilitation, University Hospital, Kyoto Prefectural University of Medicine, Kyoto, Japan; ^3^Department of Development of Multidisciplinary Promote for Physical Activity, Kyoto Prefectural University of Medicine, Kyoto, Japan; ^4^Department of Rehabilitation, Gakusai Hospital Kyoto Interdisciplinary Institute of Community Medicine, Kyoto, Japan

**Keywords:** stroke, sarcopenia, malnutrition, food texture, inflammation, older adults

## Abstract

**Background:**

Stroke-related sarcopenia (SRS) frequently emerges in the early phase of stroke and is associated with poor functional recovery and prolonged hospitalization. Identifying modifiable risk factors, such as nutritional status and food texture, may be important for early treatment in SRS. This study aimed to investigate the association between SRS and malnutrition diagnosed using the Global Leadership Initiative on Malnutrition (GLIM) criteria, with the primary aim of examining how the prevalence of SRS and malnutrition and the individual GLIM components vary among food texture levels.

**Methods:**

This study included 340 older adults (median age: 77.0 [66.0–83.0] years; median time since stroke onset: 22.0 [16.8–31.3] days) who were admitted to a convalescent rehabilitation ward during the subacute phase of stroke. SRS was diagnosed based on the Asian Working Group for Sarcopenia criteria, while malnutrition according to the GLIM framework. Food texture was categorized into three levels: standard, texture-modified, and tube feeding. Multivariate logistic regression was used to examine the association between SRS and GLIM-defined malnutrition, adjusting for relevant covariates. The Cochran–Armitage trend test assessed trends in the prevalence of SRS and malnutrition and in the proportions of individual GLIM components among food texture levels.

**Results:**

The prevalence of SRS and GLIM-defined malnutrition was 56.8 and 51.5%, respectively. Malnutrition was independently associated with SRS (odds ratio = 3.00; 95% confidence interval: 1.70–5.30; *p* < 0.001). The prevalence of both conditions increased progressively with more restrictive food textures (*p* for trend = 0.002 and <0.001, respectively). Additionally, among the GLIM components, the proportions of patients with low body mass index, reduced muscle mass, and disease burden/inflammation increased with food texture restriction (*q* for trend = 0.001, <0.001, and 0.007, respectively, after adjustment using the Benjamini–Hochberg false discovery rate correction).

**Conclusion:**

Older adults in the subacute phase of stroke who consume more restrictive food textures may be more prone to malnutrition, potentially due to stroke-related inflammation, which in turn may contribute to the development of SRS. Early tailored nutritional treatments that consider food texture restrictions and disease burden may help prevent SRS and enhance functional recovery in post-stroke rehabilitation.

## 1 Introduction

Sarcopenia, originally described as an age-related loss of skeletal muscle mass (1), is currently defined as a progressive and generalized skeletal muscle disorder characterized by the loss of muscle mass accompanied by reduced muscle strength and/or physical performance. This condition is associated with adverse outcomes, such as falls, disability, poor quality of life, and increased mortality (2, 3). The global prevalence of sarcopenia is increasing, and it is largely driven by population aging (4). Stroke is a leading cause of long-term disability among older adults, and it can also accelerate chronic process of sarcopenia. The resulting secondary sarcopenia is referred to as stroke-related sarcopenia (SRS), which is particularly prevalent in the early phase after stroke onset (5, 6). Unlike primary sarcopenia, which arises from aging, the pathogenesis of SRS is multifactorial and has not yet been fully determined (7).

Among various factors, post-stroke dysphagia requires particular attention in the onset of SRS because it may lead to complications, such as pneumonia and malnutrition (8). In the clinical setting, swallowing function is assessed early, and appropriate food texture levels are prescribed accordingly to prevent complications. These textures are typically prescribed according to the severity of dysphagia, ranging from standard diets to texture-modified diets and tube feeding. However, even when appropriate food textures are selected, a high prevalence of malnutrition in patients with stroke during the early phase and its association with poor functional outcomes have been reported (9, 10). Notably, most of these studies used nutritional screening tools or surrogate indicators, rather than standardized diagnostic criteria. To address the absence of standardized and widely accepted diagnostic criteria, the Global Leadership Initiative on Malnutrition (GLIM) proposed a consensus-based framework for the diagnosis of malnutrition in 2018. This framework incorporates phenotypic criteria and etiological criteria components (11) and has become increasingly used as a robust and internationally accepted standard for a malnutrition diagnosis.

Building on this framework, identifying modifiable factors, such as nutritional status and food texture levels, is important for understanding the development of SRS and guiding early treatment. However, evidence regarding this possibility remains limited. Only one previous study has reported associations between SRS, GLIM-defined malnutrition, and food texture levels (12), although the classification of food textures in that study was limited. Moreover, no study has comprehensively compared the distribution of individual GLIM criteria components among different texture levels. Given that most post-stroke patients experience some degree of dysphagia, food texture modification represents an inevitable and clinically relevant aspect of nutritional management. However, its role in relation to sarcopenia and GLIM-defined malnutrition has not been fully elucidated. Therefore, the primary aim of our study was to compare the prevalence of SRS and malnutrition and the proportions of individual GLIM criteria components among food texture levels. We also aimed to examine the association between SRS and GLIM-defined malnutrition. These findings may support the development of individualized nutritional treatments tailored to food texture as part of stroke rehabilitation.

## 2 Materials and methods

### 2.1 Study design and ethics

The study was a retrospective cross-sectional design, which was conducted in accordance with the STROBE guidelines and the Declaration of Helsinki, and approved by the Ethics Review Board of Kyoto Prefectural University of Medicine (ERB-C-2713-3). Written informed consent was not required because this was a retrospective study. The participants were informed about the study and provided the opportunity to opt out.

### 2.2 Participants

This study included patients in the subacute phase of stroke who were admitted to a 46-bed convalescent rehabilitation ward in Japan between February 2022 and April 2025. All patients were transferred to the rehabilitation hospital from an acute care facility after their condition had stabilized. The exclusion criteria included the presence of a cardiac pacemaker or metallic implants, loss of any of the four extremities, pregnancy, disturbance of consciousness or severe cognitive impairment, recurrent stroke, and refusal to participate.

### 2.3 Data collection

Demographic and clinical data, such as age, sex, height, weight, body mass index (BMI), type of stroke, affected side, days since stroke onset, comorbidities, dysphagia, and activities of daily living (ADL), were collected from electronic medical records. Comorbidities were assessed using the Charlson Comorbidity Index (CCI) (13). Dysphagia was assessed using the Food Intake LEVEL Scale, which is a 10-point ordinal scale of oral intake (14). ADL were assessed using the Functional Independence Measure (FIM), which includes motor and cognitive subscales (15). All assessments were performed on the day of admission.

### 2.4 SRS and GLIM-defined malnutrition diagnosis

The diagnosis of SRS was based on the Asian Working Group for Sarcopenia 2019 (AWGS 2019) criteria, which include assessments of muscle strength (grip strength) and muscle mass (skeletal muscle mass index, SMI) (3). Grip strength was measured twice on the non-affected side using a digital hand dynamometer (TKK-5401; Takei Scientific Instruments Co., Ltd., Niigata, Japan), and the highest value was recorded. Skeletal muscle mass was assessed using a bioelectrical impedance analysis with a dedicated analyzer (InBody S10; InBody Japan Inc., Tokyo, Japan). The SMI was calculated by dividing the appendicular skeletal muscle mass by the square of the patient’s height in meters. The cut-off values were as follows: SMI < 7.0 kg/m^2^ and grip strength < 28.0 kg in male; and SMI < 5.7 kg/m^2^ and grip strength < 18.0 kg in female.

Malnutrition was diagnosed according to the GLIM criteria, which involve a two-step process of initial screening and diagnostic assessment (11). The Mini Nutritional Assessment Short-Form was used for screening, with a score ≤ 11 indicating a risk of malnutrition. Patients at risk were diagnosed with malnutrition according to the presence of at least one phenotypic criterion (non-volitional weight loss > 5.0% within past 6 months or >10.0% beyond 6 months; low BMI: <18.5 kg/m^2^ if <70 years or <20.0 kg/m^2^ if ≥70 years; and reduced skeletal muscle mass assessed by a bioelectrical impedance analysis: SMI < 7.0 kg/m^2^ in male and <5.7 kg/m^2^ in female) and one etiological criterion (reduced food intake or assimilation: ≤50.0% of energy requirements for more than 1 week or any chronic gastrointestinal condition that adversely affects food assimilation or absorption; and disease burden/inflammation). In this study, disease burden/inflammation was defined as the presence of an acute disease (e.g., major infections, burns, trauma, or closed head injury) or a chronic condition (e.g., congestive heart failure, chronic obstructive pulmonary disease, rheumatoid arthritis, chronic kidney disease, chronic liver disease, or malignancy) usually associated with inflammatory activity, or as an elevated serum C-reactive protein level (>0.3 mg/dL) (11, 16).

### 2.5 Food texture levels

Food texture levels were classified into the following three groups: standard diets, texture-modified diets, and tube feeding (e.g., administration via a nasogastric tube or percutaneous endoscopic gastrostomy). Standard diets included soft and regular diets. Texture-modified diets were applied according to the Japanese Dysphagia Diet 2021, which was developed by the Japanese Society of Dysphagia Rehabilitation dysphagia diet committee using codes, such as 1j, 2–1, 2–2, and 4 (17). These codes broadly correspond to Levels 4 to 7 of the International Dysphagia Diet Standardization Initiative (18), although the Japanese Dysphagia Diet 2021 offers more detailed classifications tailored to the clinical setting in Japan.

### 2.6 Statistical analysis

All statistical analyses were performed using IBM SPSS Statistics (version 29.0; IBM Corp., Armonk, NY, United States). A two-sided *p*-value < 0.05 was considered significant. Continuous variables are expressed as the median [interquartile range] and categorical variables as percentage (number). Between-group comparisons were conducted using the Mann–Whitney *U* test for continuous variables or chi-square test for categorical variables. Multivariate logistic regression was conducted to examine the association between SRS and GLIM-defined malnutrition. Adjusted odds ratios (ORs) with 95% confidence intervals (CIs) were calculated. Covariates included age, sex, days since stroke onset, type of stroke, affected side, CCI, Food Intake LEVEL Scale, FIM motor score, and cognitive score. Multicollinearity was assessed using variance inflation factors, with values between 1 and 10 indicating no collinearity. In addition, a sensitivity analysis was performed by redefining malnutrition after excluding SMI from the GLIM phenotype. This approach aimed to isolate the influence of reduced muscle mass and to evaluate whether the observed association between SRS and GLIM-defined malnutrition was independent of this shared component. The Cochran–Armitage trend test was used to assess linear trends in the prevalence of SRS and malnutrition, as well as trends in the proportions of GLIM criteria components among food texture levels. The Benjamini–Hochberg false discovery rate correction was applied to adjust for multiple comparisons across the five GLIM components in order to control for Type I error, with statistical significance set at *q* < 0.05. A *post hoc* power analysis was conducted, limited to multivariate logistic regression, which was one of the primary analyses in this study and generally requires a larger sample size than other tests. As an exploratory analysis, a subgroup of patients who did not meet the GLIM component of reduced intake was examined to assess whether texture modification itself was associated with differences in grip strength and SMI, both of which are SRS indicators. Analysis of covariance adjusted for age, sex, BMI, and days since stroke onset was used to compare these outcomes across texture levels. Where significant group differences were detected, post-hoc tests with Bonferroni correction were conducted.

## 3 Results

**Figure 1:**
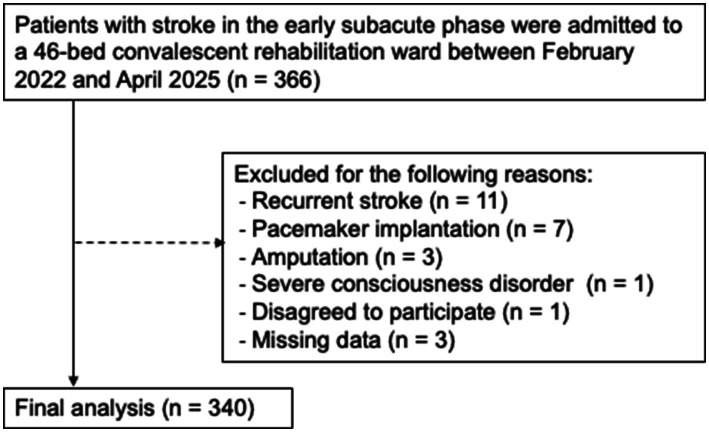
Flow diagram of the participant selection process. This figure illustrates the exclusion criteria applied during participant recruitment and the final sample analyzed.

A total of 366 patients with stroke were screened and 340 were included in the final analysis (Figure 1). Table 1 shows the demographic, clinical, and diagnostic characteristic data of the patients, presented overall and stratified by the SRS status (SRS group vs. non-SRS group). The overall median [interquartile range] age was 77.0 [66.0, 83.0] years, and the median number of days since stroke onset was 22.0 [16.8, 31.3] days, which indicated that the patients were older adults in the early subacute phase (7 days to 3 months post-onset) after stroke. The overall prevalence of SRS and GLIM-defined malnutrition was 56.8% (193 patients) and 51.5% (175 patients), respectively. Significant differences in most of the variables, including age, sex, days since stroke onset, CCI, Food Intake LEVEL Scale, FIM-motor total score, FIM-cognitive total score, and nutritional status, were observed between the SRS and non-SRS groups (all *p* < 0.050).

**Table 1:** Demographic, clinical, and diagnostic characteristics of the patients.

Patient characteristics	Overall (*N* = 340)	SRS group (*n* = 193)	Non-SRS group (*n* = 147)	*p-*value^*^
Age, years	77.0 [66.0, 83.0]	81.0 [76.0, 84.0]	68.0 [58.0, 76.0]	<0.001
Sex, % (*n*)				0.013
Male	49.4 (168)	43.5 (84)	57.1 (84)	
Female	50.6 (172)	56.5 (109)	42.9 (63)	
Height, cm	159.0 [150.5, 167.0]	155.0 [148.9, 162.0]	164.0 [156.8, 170.0]	<0.001
Weight, kg	54.2 [45.5, 63.5]	48.6 [42.6, 55.2]	62.1 [55.2, 70.8]	<0.001
BMI, kg/m^2^	21.5 [19.1, 24.1]	20.4 [18.4, 22.1]	23.6 [21.0, 25.8]	<0.001
Days since stroke onset, days	22.0 [16.8, 31.3]	23.0 [17.0, 36.0]	21.0 [16.0, 27.5]	0.035
Type of stroke, % (*n*)				0.154
Ischemic stroke	64.1 (218)	67.4 (130)	59.9 (88)	
Intracerebral hemorrhage	35.9 (122)	32.6 (63)	40.1 (59)	
Affected side, % (*n*)				0.157
Right	43.8 (149)	47.2 (91)	39.5 (58)	
Left	56.2 (191)	52.8 (102)	60.5 (89)	
Comorbidities				
Charlson’s comorbidity index	7 [6, 8]	8 [6, 9]	6 [5, 7]	<0.001
Dysphagia				
Food Intake LEVEL Scale	8 [7, 10]	7 [7, 9]	9 [8, 10]	<0.001
ADL				
FIM – motor total score	38.0 [21.0, 61.0]	29.0 [17.0, 53.0]	55.0 [28.5, 66.0]	<0.001
FIM – cognitive total score	20.5 [14.0, 27.0]	18.0 [13.0, 24.0]	24.0 [19.5, 30.0]	<0.001
Nutritional status, % (*n*)				<0.001
Malnutrition	51.5 (175)	64.8 (125)	34.0 (50)	
Non-malnutrition	48.5 (165)	35.2 (68)	66.0 (97)	
SRS indicators				
Grip strength, kg	17.8 [12.3, 25.1]	13.7 [9.5, 17.8]	27.0 [18.9, 32.8]	<0.001
SMI, kg/m^2^	5.8 [4.9, 6.7]	5.2 [4.5, 6.0]	6.7 [6.0, 7.4]	<0.001

Data are presented as the median [lower quartile, upper quartile] or percentage (number of patients). ^*^p-values were calculated between the SRS and non-SRS groups using the Mann–Whitney U test for continuous variables or chi-square test for categorical variables. SRS, stroke-related sarcopenia; BMI, body mass index; ADL, activities of daily living; FIM, Functional Independence Measure; SMI, skeletal muscle mass index.

Table 2 shows the results of the multivariate logistic regression analysis. Older age (OR = 0.92, 95% CI: 0.90–0.95, *p* < 0.001), dysphagia (OR = 1.20, 95% CI: 1.01–1.42, *p* = 0.035), and malnutrition (OR = 3.00, 95% CI: 1.70–5.30, *p* < 0.001) were independent risk factors for SRS. In the sensitivity analysis, where malnutrition was redefined after excluding SMI from the GLIM phenotype, older age (OR = 0.93, 95% CI: 0.90–0.96, *p* < 0.001) and dysphagia (OR = 1.21, 95% CI: 1.02–1.43, *p* = 0.035) remained significantly associated with SRS, whereas malnutrition was no longer significant (OR = 1.20, 95% CI: 0.66–2.17, *p* = 0.555). No multicollinearity was observed among the covariates (all variance inflation factors: <3).

**Table 2:** Clinical factors associated with stroke-related sarcopenia.

Clinical factors	Adjusted OR	95% Cl		*p-*value^*^	VIF
		Lower	Upper		
Age	0.92	0.90	0.95	<0.001	1.87
Sex	0.79	0.46	1.37	0.405	1.07
Days since stroke onset	1.00	0.98	1.02	0.704	1.10
Type of stroke	0.97	0.53	1.76	0.920	1.15
Affected side	1.69	0.96	2.97	0.069	1.08
Comorbidities					
Charlson’s comorbidity index	1.01	0.85	1.21	0.902	1.90
Dysphagia					
Food Intake LEVEL Scale	1.20	1.01	1.42	0.035	1.87
ADL					
FIM-motor total score	1.01	0.99	1.02	0.605	2.39
FIM-cognitive total score	1.03	0.98	1.09	0.276	2.43
Nutritional status	3.00	1.70	5.30	< 0.001	1.19

*p-values were obtained from the multivariate logistic regression analysis. OR, odds ratio; CI, confidence interval; VIF, variance inflation factor; ADL, activities of daily living; FIM, Functional Independence Measure; GLIM, Global Leadership Initiative on Malnutrition.

**Figure 2:**
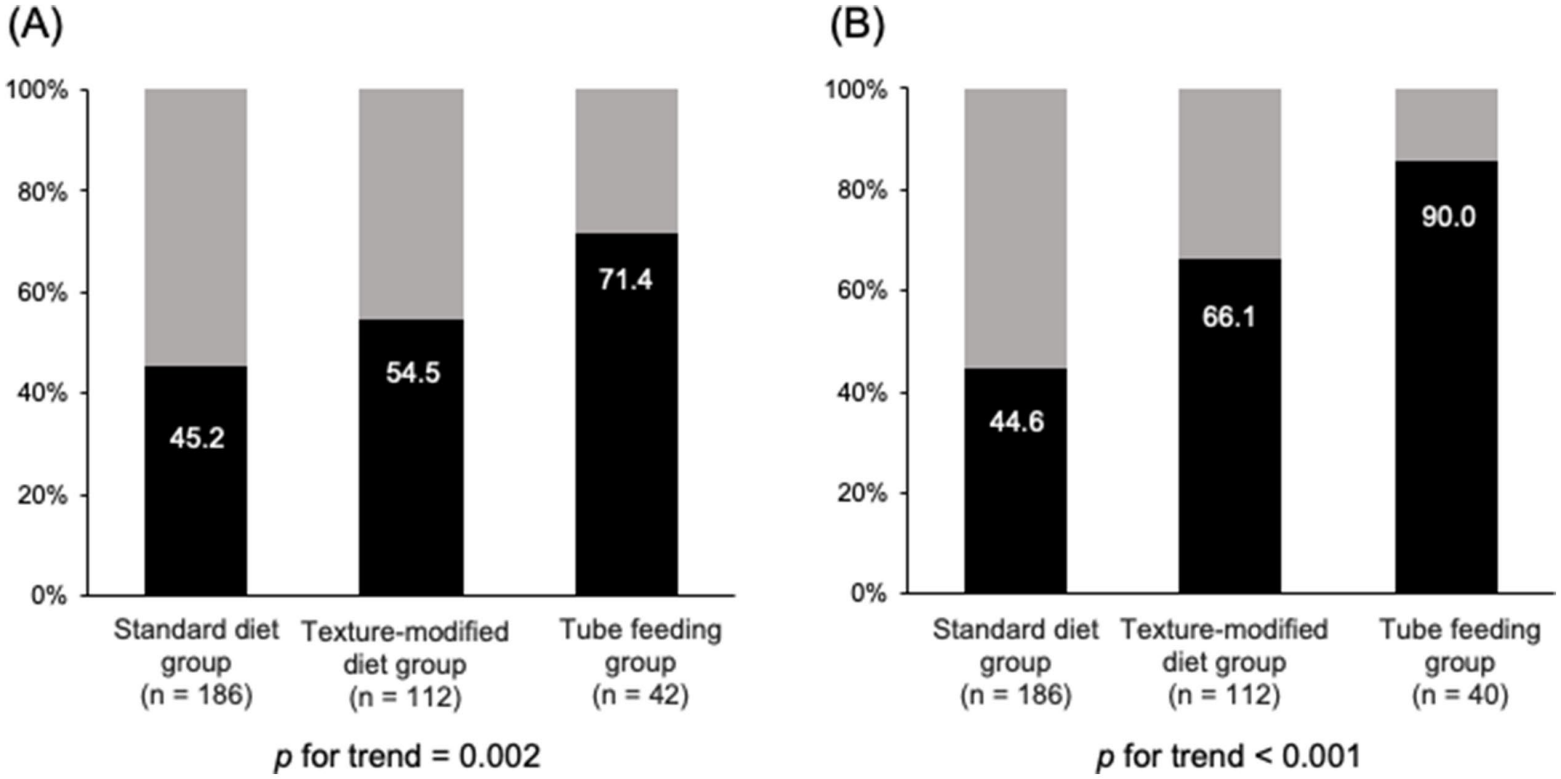
Prevalence of SRS and GLIM-defined malnutrition across food texture levels. Black bars indicate participants with SRS **(A)** or GLIM-defined malnutrition **(B)**; gray bars indicate participants without these conditions. SRS, stroke-related sarcopenia; GLIM, Global Leadership Initiative on Malnutrition.

Figure 2 shows the prevalence of SRS and malnutrition among different food texture levels. The prevalence of SRS was 45.2% in the standard diet group, 54.5% in the texture-modified diet group, and 71.2% in the tube feeding group. Similarly, the prevalence of malnutrition was 44.6, 66.1, and 90.0%, respectively. A trend in increasing prevalence was observed for SRS and malnutrition with decreasing food texture levels (*p* for trend = 0.002 and <0.001, respectively).

Table 3 shows the comparison of the individual GLIM criteria components according to the food texture levels. Significant trends were observed for a low BMI, reduced muscle mass, and disease burden/inflammation (*p* for trend < 0.001, <0.001, and 0.004, respectively), with higher proportions in the more restrictive diet groups. However, non-volitional weight loss and reduced food intake showed no significant differences between the food texture levels. After false discovery rate correction, the trend test results remained unchanged, with significant trends persisting for low BMI, reduced muscle mass, and disease burden/inflammation (*q* for trend = 0.001, <0.001, and 0.007, respectively).

**Table 3:** Comparison of GLIM criteria components among different food texture levels.

GLIM criteria components	Standard diet group (*n* = 186)		Texture-modified diet group (*n* = 112)		Tube feeding group (*n* = 42)		*p* for trend^*^	*q* for trend^†^
	Present	Absent	Present	Absent	Present	Absent		
Phenotypic criteria								
Non-volitional weight loss	33.9 (63)	66.1 (123)	31.3 (35)	68.8 (77)	35.7 (15)	64.3 (27)	0.981	0.981
Low BMI	23.7 (44)	76.3 (142)	33.9 (38)	66.1 (74)	50.0 (21)	50.0 (21)	<0.001	0.001
Reduced muscle mass	60.8 (113)	39.2 (73)	75.0 (84)	25.0 (28)	88.1 (37)	11.9 (5)	<0.001	<0.001
Etiological criteria								
Reduced food intake or assimilation	20.4 (38)	79.6 (148)	21.4 (24)	78.6 (88)	21.4 (9)	78.6 (33)	0.839	0.981
Disease burden/inflammation condition	47.8 (89)	52.2 (97)	57.1 (64)	42.9 (48)	71.4 (30)	28.6 (12)	0.004	0.007

Data are shown as the percentage (number of patients). ^*^p for trend was calculated using the Cochran–Armitage test for trend. ^†^*q* for trend (FDR-adjusted) was calculated using the Benjamini–Hochberg procedure. GLIM, Global Leadership Initiative on Malnutrition; BMI, body mass index; FDR, false discovery rate.

The *post hoc* power analysis indicated that a sample size of 340 participants was sufficient to detect an odds ratio of 3.00 with 100% power at a significance level of 0.05 (two-sided test), assuming a baseline probability of 0.5.

Table 4 shows the results of the exploratory analysis in the subgroup of patients who did not meet the GLIM component of reduced intake. Grip strength differed significantly across texture levels (*F* = 49.6, *p* < 0.001), with post-hoc tests showing significant differences among all groups (all *p* < 0.001). SMI also differed significantly across texture levels (*F* = 9.4, *p* < 0.001). Post-hoc tests showed that SMI was significantly higher in the standard diet group compared with the texture-modified diet group (*p* = 0.007) and the tube feeding group (*p* < 0.001), whereas no significant difference was observed between the texture-modified diet group and tube feeding group (*p* = 0.278).

**Table 4:** Exploratory analysis of grip strength and muscle mass among different food texture levels.

Patients who did not meet the GLIM component of reduced intake	Standard diet group (*n* = 179)	Texture-modified diet group (*n* = 107)	Tube feeding group (*n* = 42)	*p-*value^*^
SRS indicators				
Grip strength, kg	22.0 (0.5)	16.7 (0.7)	10.6 (1.1)	<0.001
SMI, kg/m^2^	6.0 (0.1)	5.7 (0.1)	5.5 (0.1)	<0.001

*p-values were calculated using analysis of covariance adjusted for age, sex, BMI, and days since stroke onset. Grip strength and SMI values are presented as estimated marginal means (standard errors). GLIM, Global Leadership Initiative on Malnutrition; SMI, skeletal muscle mass index; BMI, body mass index.

## 4 Discussion

In this study, SRS and GLIM-defined malnutrition were observed in approximately half of older patients with stroke in the subacute phase. Malnutrition was independently associated with SRS. Notably, patients who received more restrictive food textures showed a higher prevalence of both conditions. Furthermore, among the individual GLIM criteria components, the proportions of patients with a low BMI, reduced skeletal muscle mass, and disease burden/inflammation increased progressively with the severity of food texture restriction.

Previous studies that investigated patients with stroke in the early subacute phase, which is similar to the stage of patients in our study, using the Asian criteria reported that the prevalence of SRS ranged from 53.5 to 60.3% (6). In these studies, there was some variation depending on factors, such as the sample size, age, and days since stroke onset; therefore, these results should be interpreted with caution. The prevalence (56.8%) observed in our study falls within this range, supporting the notion that SRS frequently occurs in the relatively early phase post-stroke and emphasizing the importance of early screening and treatment for this condition.

Based on the hypothesis that GLIM-defined malnutrition contributes to the onset of SRS, our study examined associated risk factors in this cohort. In addition to age and dysphagia, malnutrition was significantly associated with SRS in the primary analysis. Previous systematic reviews and meta-analyses have identified a range of representative risk factors for SRS, such as age, days since stroke onset, dysphagia, and serum albumin concentrations (19, 20). Our finding that age and dysphagia were independently associated with SRS is consistent with these previous reports. While serum albumin concentrations are frequently used as a nutritional marker in the clinical setting, they are affected by non-nutritional factors and lack sensitivity to acute changes (21). To address these limitations, the present study used the GLIM criteria, which have demonstrated strong diagnostic accuracy and are increasingly adopted in the clinical setting. In our primary analysis, GLIM-defined malnutrition appeared to be independently associated with SRS; however, the sensitivity analysis excluding SMI from the GLIM phenotype attenuated this association to non-significance. This suggests that the observed relationship is largely driven by reduced muscle mass, which is a component of both diagnostic criteria. Although GLIM-defined malnutrition and SRS were treated as distinct entities in this study due to their differing conceptual frameworks, this overlap should be considered when interpreting the findings.

The prevalence of GLIM-defined malnutrition among community-dwelling older adults in Asia has been reported to be 10.7% in large-scale studies (22). In contrast, our cohort showed a prevalence of GLIM-defined malnutrition of 51.5%, highlighting the nutritional vulnerability of this population. This high prevalence reflects the combined effect of age-related factors, such as anorexia, anabolic resistance, and oral hypofunction (23), and additional stroke-related factors, which may collectively accelerate the onset of malnutrition. These findings suggest the clinical importance of comprehensive nutritional assessment in the early subacute phase after stroke.

Ensuring sufficient energy intake is a key component of nutritional management in stroke patients with malnutrition (24). To achieve this goal, food texture is modified according to the severity of dysphagia in the clinical setting. In this study, the prevalence of SRS and malnutrition showed a significant increasing trend across patient groups with more restrictive food texture levels. A previous study indicated that patients who received tube feeding often met energy requirements more consistently than those on oral diets (25). Similarly, texture-modified diets often include added fluids, which increase the volume of intake, and have been reported to result in a lower total energy intake than regular diets (26). Although energy intake is theoretically higher in the order of tube feeding group > standard diet group > texture-modified diet group, the observed prevalence of SRS and malnutrition were highest in the order of tube feeding group > texture-modified diet group > standard diet group. This finding suggests that factors beyond energy intake may contribute to the onset of SRS triggered by malnutrition. In addition, our exploratory subgroup analysis of patients who did not meet the GLIM component of reduced intake demonstrated that more restrictive food texture levels were associated with lower grip strength and modestly lower SMI. These findings suggest that texture modification may reflect muscle weakness or loss of muscle mass independent of reduced dietary intake. This highlights the importance of considering food texture levels as a potential risk factor for both malnutrition and SRS.

This study further analyzed the phenotypic and etiological components of the GLIM criteria among different food texture levels. Regarding the phenotypic components, patients on more restrictive food textures showed a significant increasing trend in the proportions of a low BMI and reduced skeletal muscle mass, while non-volitional weight loss did not follow this trend. Non-volitional weight loss is considered a dynamic criterion and may be masked by fluid retention or by nutritional support via tube feeding (27). In contrast, a low BMI and reduced muscle mass are static indicators and may be more readily apparent, especially in older adults with low baseline values. These findings suggest that a low BMI and muscle mass may be more practical markers for early malnutrition than recent weight loss in this patient population.

Regarding the etiological criteria, disease burden/inflammation was significantly more frequently observed in patients with more restrictive diets, whereas such a trend was not observed for a reduced intake or malabsorption. In the early phase after stroke, inflammatory responses triggered by brain injury contribute to deterioration of the nutritional status through increased energy demands and protein catabolism (28). This inflammatory response may be further aggravated in severely ill patients who require tube feeding or texture-modified diets because these individuals are particularly susceptible to infections, including but not limited to pneumonia (29, 30), thereby potentially accelerating the progression of malnutrition.

Generally, energy requirements are estimated in the clinical setting by multiplying basal energy expenditure by activity and stress factors (31). Although nutritional treatment is recommended to be intensified in clinical guidelines (32), the stress component is often omitted in hospitalized patients with stroke because it is generally considered unnecessary (33). However, a recent study showed that energy-enriched, texture-modified diets contributed to improved ADL in older patients with stroke and dysphagia (34). This result is consistent with the 2018 guideline of the European Society for Clinical Nutrition and Metabolism on clinical nutrition in neurology, which recommends individualized, texture-adapted food treatments for such patients (35). Therefore, our findings suggest that an inflammatory response related to stroke plays a critical role in deterioration of the nutritional status, highlighting the need for targeted nutritional treatments in this high-risk population. Nevertheless, the retrospective and cross-sectional design of the study limits the ability to infer causality. Future longitudinal studies are warranted to validate the effectiveness of personalized nutritional treatments in this vulnerable population.

This study has several limitations. First, the cross-sectional nature of the study limited the ability to draw causal inferences between SRS, malnutrition, and food texture levels. Second, this study was conducted in a single rehabilitation hospital in Japan, which may limit the generalizability of the findings to other settings or populations. Third, pre-stroke sarcopenia was not assessed, causing difficulty in distinguishing new-onset SRS from pre-existing sarcopenia. Fourth, reduced muscle mass is a shared diagnostic component of both SRS and GLIM-defined malnutrition, which may have partially contributed to the observed association. Although this issue was further examined in a sensitivity analysis, the overlap between the criteria remains a limitation. Nevertheless, the GLIM criteria are currently the most widely accepted and internationally recognized standard for diagnosing malnutrition and were therefore considered appropriate for use in this study. Finally, although a serum C-reactive protein level was incorporated into the operational definition of disease burden/inflammation, other direct biomarkers such as interleukin-6 were not measured. In addition, precise dietary intake data, exact quantification of macronutrient and micronutrient content, and the duration of each texture level were unavailable. These omissions may have left residual confounding unaddressed.

## 5 Conclusion

This study showed that GLIM-defined malnutrition was independently associated with SRS in older patients during the early subacute phase after stroke. The prevalence of both conditions was higher with increasing food texture restriction, and specific GLIM criteria components, such as a low BMI, reduced muscle mass, and disease-related inflammation, were more frequently observed in patients with more restrictive food textures. These findings suggest that food texture levels may serve as a potential indirect marker of nutritional status (e.g., low BMI, reduced muscle mass) and factors such as inflammation in the early phase after stroke. Early rehabilitation treatment that incorporates personalized nutritional management tailored to food texture may help mitigate malnutrition, prevent SRS, and improve functional outcomes in this population.

## Data Availability

The datasets generated and analyzed during the current study are not publicly available due to patient privacy and institutional regulations, but are available from the corresponding author upon reasonable request. Requests to access the datasets should be directed to Norikazu Hishikawa, hisikawa@koto.kpu-m.ac.jp.
